# Real-World Use of Extended Half-Life Factor IX in Children with Haemophilia B

**DOI:** 10.3390/life15091352

**Published:** 2025-08-26

**Authors:** Athina Dettoraki, Aikaterini Michalopoulou, Stefanos Saslis, Konstantina Dakou, Ioanna Stamati, Sotiria Thymianou, Zoey Kapsimali, Olympia Papakonstantinou, Helen Pergantou

**Affiliations:** 1 Haemophilia Centre, Haemostasis and Thrombosis Unit, “Aghia Sophia” Children’s Hospital, 1 Thivon and Papadiamantopoulou Str., 115 27 Athens, Greece; katia.michalopoulou@gmail.com (A.M.); stefan_sasli_s@hotmail.com (S.S.); kos.dakou@gmail.com (K.D.); istamati71@gmail.com (I.S.); sothym@gmail.com (S.T.); zoeykapsimali@gmail.com (Z.K.); hpergantou@gmail.com (H.P.); 2 2nd Department of Radiology, National and Kapodistrian University of Athens, “Attikon” University Hospital of Athens, 1 Rimini Str., Chaidari, 124 62 Athens, Greece; sogofianol@gmail.com

**Keywords:** haemophilia, prophylaxis, extended half-life factor concentrates, standard half-life factor concentrates, annual bleeding rate, annual joint bleeding rate

## Abstract

Introduction Limited studies have reported data on the real-world clinical use of extended half-life (EHL) IX products in children and adolescents with haemophilia B, also referred to as people with haemophilia B (PWHB). Aim To examine the real-life experience with EHL factor IX products in PWHB regarding the clinical experience with standard half-life products (SHL). Methods A retrospective review of medical records of PWHB who have been prescribed EHL IX factor concentrates was conducted. Results Fourteen male PWHB were enrolled in the study, all with severe bleeding phenotypes being on prophylaxis (1–6 years old: *n* = 3, 7–12 years old: *n* = 5 and 13–18 years old: *n* = 6). Four of them were previously untreated patients; nine out of fourteen children (64%) had severe, four had moderate, and one child had mild haemophilia B. Median length of follow-up was 45 months (range 16–84 months). Children who transitioned from SHL prophylaxis to EHL prophylaxis experienced changes in their treatment outcomes. The median dosing interval increased from 3.5 days to 7 days, mean trough levels rose from 4.3% to 15.3% among children with severe haemophilia B, and the mean annual bleeding rate (ABR) decreased from 1.8 to 1 (*p* = 0.3, Wilcoxon test). Significant differences were found in EHL vs. SHL use regarding (a) the factor IX consumption for prophylaxis and bleeds (*p* = 0.046, *t*-test), with the EHL consumption (45.6 IU/kg/week) being significantly lower than the SHL consumption (70.3 IU/kg/week), and (b) the factor IX consumption only for prophylaxis (*p* = 0.022, *t*-test), with the EHL consumption (37.0 IU/kg/week) being significantly lower than the SHL consumption (66.0 IU/kg/week). There was no inhibitor development. Conclusion This study demonstrates the successful use of EHL prophylaxis in PWHB.

## 1. Introduction

Patients with moderate to severe haemophilia B face significant risks of developing serious complications, including frequent bleeding episodes and recurrent joint bleeds. The standard of care for individuals with severe and sometimes moderate haemophilia B is prophylactic treatment using factor IX concentrates [1]. For PWHB, optimal prophylaxis with standard half-life factor concentrates (SHL) necessitates two weekly infusions. To extend the half-life of factor IX and enhance prophylactic treatment, reducing the frequency of infusions, several extended half-life (EHL) factor IX concentrates have been developed. Numerous studies [2,3,4,5] indicate that EHL concentrates are both well tolerated and demonstrate effectiveness.

This study provides a comparison of EHL and SHL factor concentrate prophylaxis regimens in paediatric and adolescent patients with haemophilia B, based on real-world data from our centre. Also, prophylaxis with EHL-FIX in Previously Untreated Patients (PUPs) with haemophilia B is recorded.

## 2. Materials and Methods

### 2.1. Study Population

A retrospective analysis of medical records for patients with haemophilia B who underwent treatment with EHL factor concentrates was conducted in our centre. The centre manages a total of 130 patients with various types of haemophilia, including 25 with haemophilia B, aged from 1 month to 18 years. To clarify, 11 children with haemophilia B are not in the study because two of them have severe haemophilia B with high-responding inhibitors being on a pharmaceutical protocol with subcutaneous non-factor therapy, one has severe haemophilia and is on extended half-life (EHL) prophylaxis but for a short period, less than one year, and one has severe haemophilia and is on prophylaxis with a standard half-life (SHL) product, while 3 with moderate haemophilia and 4 with mild disease, both with mild bleeding phenotypes, are not on prophylaxis.

### 2.2. Study Design

The medical records of each subject were reviewed to determine the following data: age, gender, ethnicity, race, type and severity of haemophilia, previous treatment in switchers and dosing regimen, reason for switch, current dosing regimen, trough levels, in vivo recovery, pharmacokinetics via WAPPS-Hemo (Web Accessible Population Pharmacokinetic Service-Haemophilia), Annual Bleeding Rate (ABR) and Annual Joint Bleeding Rate (AJBR), HJHS (Haemophilia Joint Health Score) [6], average factor consumption for prophylaxis in IU/kg/week, total consumption data including bleeds, development of inhibitors and non-bleeding adverse events. Also, relative data for PUPs with haemophilia B were recorded. Total consumption information, including bleeding episodes 12 months prior and 12 months after EHL treatment utilisation was tabulated. Patients switching to or starting PUPs EHL concentrates underwent pharmacokinetic studies, according to our clinical protocol. Peak factor levels were reported as In vivo Recovery (IR, IU/dL/IU/Kg) [7]. Plasma levels of factor IX clotting activity (FIXc) were measured using one-stage clotting assays for rFIX-Fc and rFIX-FP concentrates, while chromogenic assays were used for FIX-pegylated products. To be more precise, PK assessments for FIX activity were primarily based on an activated Partial Thromboplastin Time (aPTT)-based one-stage clotting assay performed on a coagulation analyser (Werfen ACLTOP750^CTS^ analyser) [WERFEN, Instrumentation Laboratory Company, USA], using *SynthASil* (HemosIL, Instrumentation Laboratory Company, Bedford, USA-Milano, Italy) as an aPTT reagent, which is acceptable for measurements on rFIX-Fc and rFIX-FP.

No child had a prior FIX inhibitor history. A positive inhibitor result was defined as a neutralising antibody value ≥ 0.6 Bethesda units/mL confirmed by Bethesda assay within 4 weeks of the initial occurrence [8]. Data were reported as means and ranges, and all prior/after comparisons were performed with the Wilcoxon matched pairs signed ranks test. Patients from across Greece, mainly Athens, were followed exclusively at the Athens Paediatric Haemophilia Centre from January 2018 to December 2024.

## 3. Results

### 3.1. Patient Demographics

A total of 14 subjects (60% of the total of patients with haemophilia B in our Centre) (1–6 years old: *n* = 3, 7–12 years old: *n* = 5 and 13–18 years old: *n* = 6) with haemophilia B were enrolled in the study. Of the 14 children, 9 (64%) had severe haemophilia B (median age 11, range 2.5–18 years), 4 had moderate (median age 12.7, range 8.4–15.1 years), and 1 had mild haemophilia B (age 18) with a severe bleeding phenotype. All subjects are Caucasian; none have inhibitors. Ten patients (switchers) had been previously treated with recombinant FIX concentrates for >100 EDs (Exposure Days). Three toddlers and a child (all PUPs with severe haemophilia B) started prophylaxis with extended half-life products.

Eight out of ten children (80%) with haemophilia who switched to EHL (switchers) had been previously on a prophylaxis regimen; four of them had severe and the remainder four had moderate haemophilia B. Two patients, one with moderate and one with mild haemophilia B, who had been previously on on-demand therapy, switched to prophylaxis with EHL products due to severe bleeding phenotype. It is emphasised that four PUPs started prophylaxis on EHL products. Of the ten PWHB switchers, five switched to recombinant coagulation FIX fusion with albumin (FIX-FP), three to recombinant coagulation FIX fusion protein Fc (FIX-Fc) and two to pegylated recombinant coagulation FIX (PEG-polyethylene glycol-IX). Four previously untreated patients (PUPs) began prophylactic treatment: two received FIX-FP and two FIX-Fc. The median duration of treatment with FIX EHL products was 45 months, with a range from 16 to 84 months.

### 3.2. Reason for Switch

The reason for switching was to “improve quality of life” in eight out of ten (80%) of children and to “improve compliance” in two of them (20%).

### 3.3. Children with Severe Haemophilia Switching to EHL Products (Comparing Prophylaxis on SHL Products)

#### 3.3.1. Trough Levels

**Figure 1:**
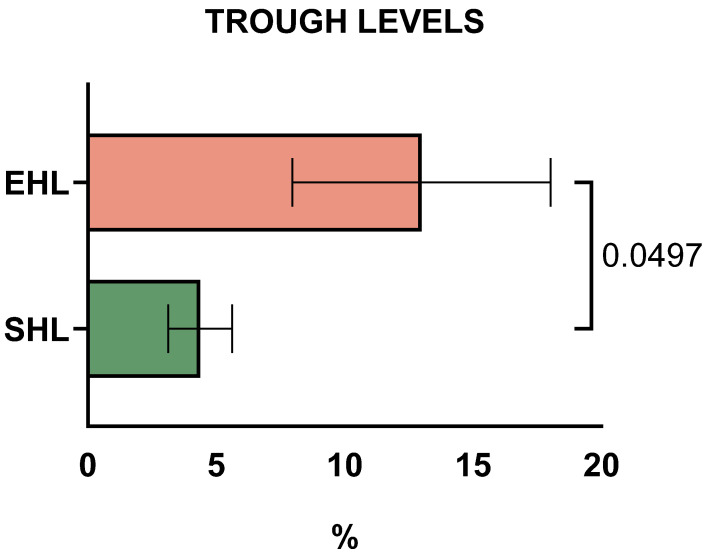
Trough levels of standard half-life (SHL) and extended half-life (EHL) factors in children and adolescents with severe haemophilia B (PWHB), during SHL and EHL treatment with Factor IX, respectively.

Mean FIX trough levels were 15.3% (range: 8–20%) in PWHB on EHL concentrates (three children on rFIX-FP, one on rFIX-Fc) versus 4.3% (range: 3–6%) on SHL concentrates (Figure 1, *p* = 0.0497). For the three patients with severe haemophilia B, mean trough FIX levels were 14.6% (range: 12–20%) on rFIX-FP and 4.3% (range: 3–6%) on SHL concentrates.

#### 3.3.2. In Vivo Recovery

Mean in vivo recovery was 1.35 IU/dL/IU/kg (range: 1.2–1.5 IU/dL/IU/kg) for EHL prophylaxis, while it was 0.9 IU/dL/IU/kg (range: 0.7–1.02 IU/dL/IU/kg) for SHL FIX prophylaxis. For the three patients with severe haemophilia B, mean in vivo recovery of rFIX-FP was 1.35 IU/dL/IU/kg (range: 1.2–1.5), while SHL concentrates showed a recovery of 0.91% (range: 3–6%).

#### 3.3.3. WAPPS Estimates

For all, apart from two PWHB, data were workable on EHL estimated balanced half-time, calculated using WAPPS-Hemo, a web-accessible, pharmacokinetic modelling database.

Among patients with severe haemophilia B who transitioned to EHL products, the mean half-life was 140 h (range: 93.25–183.75 h). On SHL prophylaxis, the mean half-life was 45 h (range: 37.75–56.5 h). The median post-infusion time to 5% estimate was 15 days (range: 9.5–24 days) and to 10% estimate was 11.5 days (range: 6–17 days) on EHL products.

#### 3.3.4. Mean Factor Consumption for Prophylaxis and Bleeds

**Figure 2:**
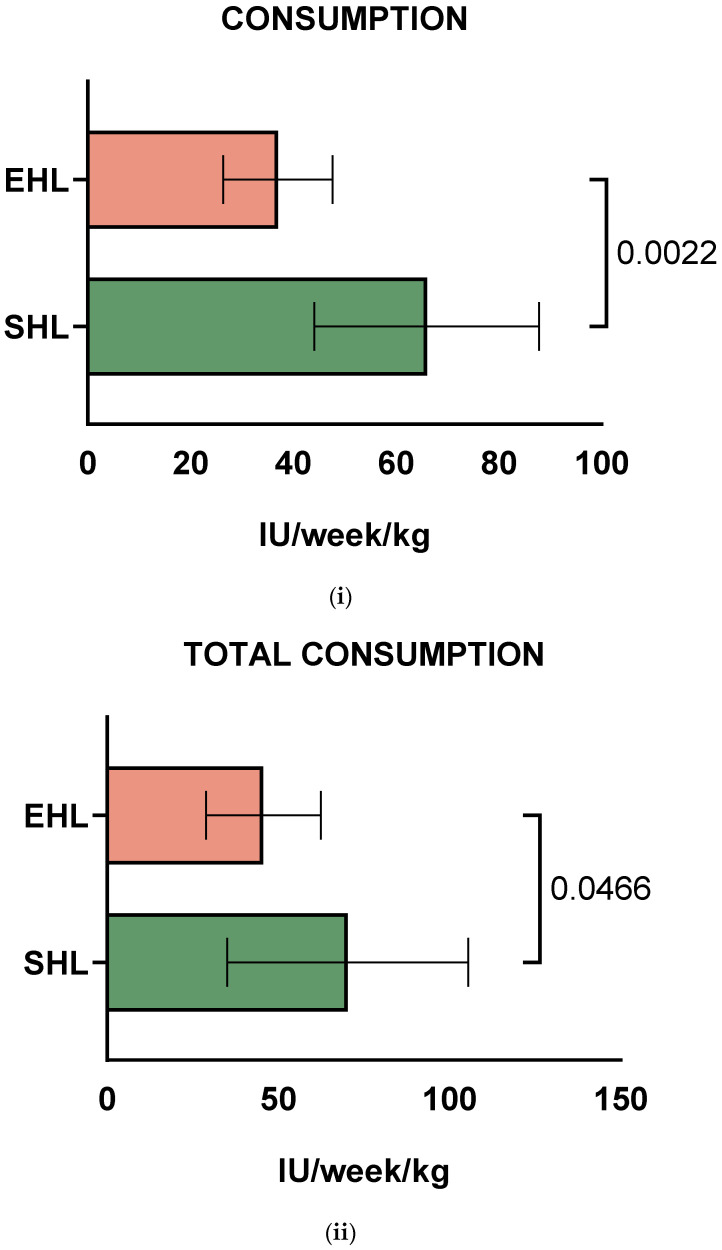
Average factor consumption values for (**i**) prophylaxis and (**ii**) total consumption (including prophylaxis and bleeds) for children and adolescents with severe haemophilia B (PWHB), during standard half-life (SHL) and extended half-life (EHL) treatment with Factor IX, respectively.

Data on both SHL and EHL weekly consumption for all 10 PWHB who switched were available. The mean factor consumption values for prophylaxis were 66 IU/kg/wk (range: 29–128 IU/kg/wk) for SHL FIX products and 37 IU/kg/wk (range: 14.5–46 IU/kg/wk) for EHL FIX products (*p* = 0.0022, Figure 2i), showing that transition from SHL to EHL decreased factor consumption due to prophylaxis by 44%. Interestingly, the pairing was significantly effective (correlation coefficient r = 0.79, correlation *p*-value = 0.0172). It should be noted that EHL consumption levels were lower than for the SHL concentrates for all PWHB. After switching to EHL, total factor consumption, including bleeds, was significantly decreased in PWHB [70.3 IU/kg/week (range: 50–104 IU/kg/wk) vs. 45.6 IU/kg/week (range: 34–77.5 IU/kg/wk), *p* = 0.0466, Figure 2ii]. Also, the pairing was significantly effective (correlation coefficient r = 0.71, correlation *p*-value = 0.037).

### 3.4. Dosing Regimen

Regarding the dosing regimen for prophylaxis, EHL treatment required less frequent infusions than the corresponding SHL treatment. For all PWHB who were on prophylaxis and switched to EHL, the average number of infusions per week was reduced from 2 to 1 after initiation of EHL concentrates. The median dosing interval lengthened from 3.5 days to 7 days (*p* = 0.02). The mean dose per injection increased from 39.6 IU/kg to 44.7 IU/kg (*p* > 0.5) for PWHB, not statistically significantly. Specifically, for children and adolescents with severe haemophilia B on rFIX-FP, the mean dose per injection increased from 34.8 IU/kg to 41.3 IU/kg (*p* > 0.5).

### 3.5. ABRs and AJBRs

ABRs, of course, remained low before and after switching to EHL concentrates in comparison with past years and were constantly lower in patients on EHL prophylaxis. Bleeding history was collected one year prior to the initiation of EHL treatment and one year after transition. No PWHB had target joints. The ABR was 1.8 (range 0–4) in the 12 months before the switch and decreased to 1 (range 0–2) in the following 12 months (*p* = 0.3). The mean AJBR was 0.7 (range: 0–1) for the 12 months before the switch and stayed at 0.7 (range: 0–2) for the 12 months after (*p* = 0.6).

### 3.6. HJHS

From the beginning to the end of the study, the mean changes in HJHS 2.1 [6] were zero for PWHB. Mean HJHS 2.1 remained the same (0.2) at the end of the first year of switching to EHL, being very low though.

### 3.7. Reported Bleeds

Of the fourteen children with haemophilia B who received FIX EHL products, four (29%) experienced bleeding episodes, while two of them (2/4) did not have any joint bleeds. They had a total of ten bleeding episodes (five joint bleeds), all post-traumatic. One patient experienced a hip joint bleed while on FIX EHL that occurred very early at the time of initiation of EHL concentrate (37 IU/Kg—a relatively lower dose than that indicated). The other boy that had joint bleeds was a Greek teenage champion swimmer who had a traumatic right knee bleed on EHL. Notably, a 6-year-old male patient with severe haemophilia B experienced five episodes of muscle haematomas in the lower extremities (calf muscles) despite maintaining high trough FIX levels, leading to a transition to an alternative extended half-life FIX product. The child received prophylaxis with the new EHL-FIX, resulting in fewer bleeds. All calculations and parameters in this study were based on the child’s treatment with the new EHL-FIX. Five of the ten total bleeds were from a single outlier patient, so data on his previous regimen were excluded. We did not include these outlier bleeds in ABRs.

### 3.8. Children with Moderate Haemophilia Switching to EHL-FIX

There were four patients with moderate haemophilia B, one of them previously treated on demand. Trough FIX levels of the children with moderate disease who switched to EHL products was 6.5% (range: 6–7%) on EHL prophylaxis versus 3.3% (range: 2–6%) on SHL prophylaxis, while mean in vivo recovery was 1.1 IU/dL/IU/kg (range: 0.7–1.5 IU/dL/IU/kg) on EHL prophylaxis versus 0.7 IU/dL/IU/kg (range: 0.6–1.01 IU/dL/IU/kg) on SHL prophylaxis. The EHL balanced half-time, calculated using WAPPS, was 112.75 h (range: 67.25–158.25 h) on EHL prophylaxis and 28.6 h (range: 28.5–28.75 h) on SHL prophylaxis. The median post-infusion time to 5% estimate was 14.5 days (range: 9–19 days) on EHL prophylaxis and 2.7 days (range: 1.1–4.3 days) on SHL prophylaxis. The mean total factor consumption values for EHL prophylaxis were 51 (IU/kg)/wk [range: 34–51 (IU/kg)/wk], whereas for SHL prophylaxis they were 62 (IU/kg)/wk [range: 32–116 (IU/kg)/wk]. The patients were dosed weekly. The mean ABR and AJBR were 1.3 (range: 0–1) and 1.2 (range: 0–1), respectively.

### 3.9. Starting Prophylaxis with EHL-FIX in PUP

Three toddlers and one child (all with severe haemophilia) started prophylaxis with an extended half-life product. The median length of time that patients were treated with FIX EHL products was 21 months (range 19–33 months). The number of infusions per week was one in all children, and the mean dose per injection was 57.3 IU/kg (range: 45.5–66.7 IU/kg). Mean trough FIX levels (N = 4) were 10% (range: 6–15%), and mean in vivo recovery was 0.965 IU/dL/IU/kg (range: 0.8–1.22 IU/dL/IU/kg) for EHL prophylaxis. No serious bleeds were reported. The mean half-time was 103.25 h (range: 81.75–128.25 h). The median post-infusion time to 5% estimate was 12.9 days (range: 8.8–16.6 days).

### 3.10. Inhibitors and Adverse Events

All children remained inhibitor-free being on FIX EHL concentrates. None of the patients experienced non-bleeding adverse events while receiving EHL FIX treatment or discontinued EHL FIX treatment.

## 4. Discussion

Regular prophylactic treatment in haemophilia aims to prevent bleeding episodes, and it is the goal of therapy to preserve normal musculoskeletal function. Eight years ago, prophylactic treatment with EHL concentrates was introduced in 60% of all PWHB followed at our Centre. It led to fewer injections, improved treatment adherence in PWHB, much higher trough levels and improved bleeding outcomes. To improve quality of life was the main motivation for changing treatment from SHL to EHL factor concentrates.

In our centre, patients with haemophilia are encouraged to record factor infusions and bleeding events requiring treatments in paper or electronic diaries as well as treatment protocols. From these sources, the following information was extracted: number and dates of infusions and amount (in International Units-IU) of factor product, intention of infusion (prophylactic or on-demand treatment) and nature (spontaneous or traumatic) and location of bleeding. Furthermore, information on patients’ prescribed factor products is collected by our dedicated haemophilia nurses. In this way, compliance and adherence could be concluded. Nonetheless, most patients and their parents usually complete the HaemoQuol questionnaire according to age, so useful conclusions could be extracted from them. Quality of life could also be measured by the number of infusions, as in patients on extended FIX products the median dosing interval was significantly lengthened from 3.5 to 7 days. 

In addition, there was a numerical decrease without statistical significance in ABR or AJBR for PWHB when 12 months preceding the switch and 12 months after the switch were considered. Mean HJHS remained the same (0.2) at the end of the first year of switching to EHL, being very low though.

Some results from our study seem to be similar to the published clinical trials and “real life” publications in other centres. Firstly, the median interval between prophylaxis injections increased from 3.5 to 7 days for PWHB on EHL prophylaxis, similar to results in older patients [2,4]. Secondly, we noted low ABR and AJBR for PWHB on EHL prophylaxis, as has been already discussed. To be more precise, mean ABR in PWHB was 1.8, which fell to 1, while AJBR remained stable (0.7), following treatment with EHL concentrates. Increased adherence, longer lifetime at higher factor levels and achievement of higher trough levels could definitely contribute to better bleeding outcomes [9,10,11]. However, in one child a bleeding event occurred very early at the time of initiation of EHL FIX concentrate with a dose not yet adjusted. Another child suffered five leg muscle haematomas despite having high trough FIX levels (>15%) and was categorised as an outlier patient; he was subsequently switched to a different EHL-FIX. He had a notable breakthrough bleeding tendency on one preparation that improved after switching to another. Similar reports exist in the literature [12]. Likewise, data by Powell JS [4] showed that older haemophilia B patients on EHL rFIX-Fc had median ABRs at 3. For one child in our study, only the first 16 months on EHL prophylaxes were assessed, and it is possible that EHL dosing was not adjusted to bleeding phenotype and pharmacokinetic results. Furthermore, no statistically significant decrease in ABRs in our study may be due to the small number of PWHB. Perhaps better trough levels offer a sense of safety and increase physical activity, which may result in increased bleeds in some children. Notably, our real-world data show a reduction in ABR even in a population that already had low ABRs on SHL, indicating that EHL FIX may offer superior bleed protection in clinical practice. Trough levels were substantially higher with EHL, offering a potential explanation for improved outcomes. 

Moreover, it is noteworthy that patients who transitioned to the EHL FIX product versus prior years’ SHL prophylaxis reduced their factor consumption for prophylaxis, a consistent finding across studies [13,14,15,16,17,18]. Following the transition to EHL, there was a reduction in total factor consumption, including bleed-related usage, among PWHB. This decrease has positively influenced the burden experienced by patients and caregivers, as reported by parents during annual haemophilia assessments—a finding that aligns with observations documented in the literature [15]. Furthermore, the absence of inhibitor development supports the safety of EHL products in the paediatric population. Finally, none of our patients returned back to SHL products or even stated wanting to return back to their prior treatment.

Also, PUPs with severe haemophilia B demonstrated excellent tolerance (no adverse events) and efficacy (no serious bleeds) being on EHL prophylaxis and, furthermore, reduced treatment burden (once-per-week infusions), as it is in concordance with the current literature [19,20,21].

One strength of this study is the relatively long follow-up period, with some patients monitored for up to eight years. This provides valuable longitudinal insight into the sustained benefits of EHL prophylaxis.

There are several limitations in our study, though. Firstly, there was not a specified protocol to switch due to its retrospective design. Secondly, selection of patients for switching to EHL factors may have been subject to bias. Thirdly, the sample size (fourteen patients) was small. However, while the sample size is small, the study includes patients across all paediatric age ranges and disease severities. The study is also from a single centre, which may limit generalisability. Nevertheless, the consistency of findings with published trial data supports the external validity of the results. Finally, the population is highly heterogeneous: it includes patients with different disease severities, treatment histories prior to switching to EHL, and different EHL products. This makes it very difficult to draw generalisable conclusions. EHL-FIXs vary in PK properties such as volume of distribution, incremental recovery, extravasal distribution, and plasma levels, which should be considered. Acknowledging the limitations, we believe that our ‘real-life’ study may provide important clinical data results for Greek patients with haemophilia B, a very rare disease.

## 5. Conclusions

This retrospective cohort study demonstrates successful prophylaxis of PWHB using EHL FIX concentrates, with benefits including reduced infusion frequency, improved bleeding outcomes, and lower FIX usage. No inhibitors were observed, supporting the safety of EHL products. EHL prophylaxis should be considered for PWHB to improve treatment adherence and quality of life. PK-guided dose optimisation may further reduce consumption while maintaining bleed protection. Additional real-world data from larger cohorts and other EHL FIX products will further inform best practices in the management of paediatric haemophilia B.

This retrospective cohort study demonstrates successful prophylaxis of CWHB using EHL FIX concentrates, with benefits including reduced infusion frequency, improved bleeding outcomes, and lower FIX usage. No inhibitors were observed, supporting the safety of EHL products. EHL prophylaxis should be considered for CWHB to improve treatment adherence and quality of life. PK-guided dose optimisation may further reduce consumption while maintaining bleed protection. Additional real-world data from larger cohorts and other EHL FIX products will further inform best practices in the management of paediatric haemophilia B.

## Data Availability

The data presented in this study are available on request from the corresponding author.
